# Rapid Pulsed Light Sintering of Silver Nanowires on Woven Polyester for personal thermal management with enhanced performance, durability and cost-effectiveness

**DOI:** 10.1038/s41598-018-35650-7

**Published:** 2018-11-21

**Authors:** Hyun-Jun Hwang, Harish Devaraj, Chen Yang, Zhongwei Gao, Chih-hung Chang, Howon Lee, Rajiv Malhotra

**Affiliations:** 1 Department of Mechanical and Aerospace Engineering, Rutgers University, 98 Brett Road, Piscataway, New Jersey 08854 USA; 2 School of Chemical, Biological and Environmental Engineering, Oregon State University, Johnson Hall, Suite 216, Corvallis, Oregon 97331 USA

## Abstract

Fabric-based personal heating patches have small geometric profiles and can be attached to selected areas of garments for personal thermal management to enable significant energy savings in built environments. Scalable fabrication of such patches with high thermal performance at low applied voltage, high durability and low materials cost is critical to the widespread implementation of these energy savings. This work investigates a scalable Intense Pulsed Light (IPL) sintering process for fabricating silver nanowire on woven polyester heating patches. Just 300 microseconds of IPL sintering results in 30% lesser electrical resistance, 70% higher thermal performance, greater durability (under bending up to 2 mm radius of curvature, washing, humidity and high temperature), with only 50% the added nanowire mass compared to state-of-the-art. Computational modeling combining electromagnetic and thermal simulations is performed to uncover the nanoscale temperature gradients during IPL sintering, and the underlying reason for greater durability of the nanowire-fabric after sintering. This large-area, high speed, and ambient-condition IPL sintering process represents an attractive strategy for scalably fabricating personal heating fabric-patches with greater thermal performance, higher durability and reduced costs.

## Introduction

47% of global energy today is spent on indoor heating and nearly 42% of this energy is wasted to heat empty space and inanimate objects rather than humans^1^. Thus, reducing the energy consumption for indoor heating is a crucial part of solving the global energy crisis^2,3^. An emerging paradigm to resolving this issue is personal thermal management, i.e., lowering building heating needs by focusing on heating the human body as per individual needs. An effective means of realizing this approach is a low-profile Joule heating unit that can be pre-sewed on as patch to the clothing we wear near regions of the body where we feel the coldest^4–10^. One approach to creating such patches is to directly coat existing fabrics with conductive nanomaterials, such as graphene^11,12^, carbon nanotubes (CNTs^13,14^), and metallic nanowires. This coating can be performed via solution approaches such as dip-coating and spray-coating of nanomaterial inks directly on to the fabric. This has advantages over other approaches such as weaving pre-coated fibers^12^ which causes coating degradation, using polymer substrate based patches^15^ which increases cloth stiffness, or using hard-to-scale vacuum deposition processes^12^.

Electrically conductive active fabrics have seen significant interest recently^16–18^. The solution approach has been used to coat graphene and carbon nanotubes onto fabrics because of their inherently high electrical conductivity and flexibility. Unfortunately, these nanomaterials exhibit high sheet resistance (100–1000 ohm·sq^−1^) after deposition. This is because the junction electrical resistance between the deposited nanomaterials is typically quite high^19^, resulting in high electrical resistance of the patch and low thermal performance during joule heating. Overcoming this issue often requires complex chemical processing steps or high temperature annealing which can damage the underlying fabric. Metal nanowires have attracted considerable attention as a promising alternative^7,8,20,21^ with silver nanowires being particularly attractive because they are cheaper than gold nanowires and more stable to corrosion and oxidation than copper^22,23^. Further, if the average inter-nanowire spacing is lesser than the wavelength at which the body radiates heat (≈9 µm) then the silver nanowire-coated-fabric also reduces thermal losses from the body while retaining fabric breathability due to porosity of the nanowire network^7^. Moreover, such Ag nanowire networks also exhibit antibacterial properties^24^ and UV-blocking properties^25^. It has been estimated that such Ag nanowire-fabric heating patches can annually save up to 1000 kWh of the energy used to heat the built environment, per person, when used for personal thermal management^7^.

The maximum temperature rise required during heating from such personal heating patches is typically 35 °C^26^. The relationship between thermal power generation and electrical resistance of such patches is expressed as *Q* = *V*^2^*/R*, where *Q* is the thermal power generated, *V* is the applied voltage, and *R* is the electrical resistance of the patch. High electrical resistance results in greater voltage needed to attain the above temperature rise, and thus low electrical resistance of the patch is highly desirable. Dip-coating and drying at low temperatures is a common method for fabricating Ag nanowire-fabrics, with a reduction in resistance being achieved by increasing the nanowire concentration coated onto the fabric^7,8^. However, this approach also increases the patch cost due to increased total materials cost of nanowires used.

Sintering or annealing of the deposited nanowires, resulting in fusion at the nanowire contact points, is an alternative way to reduce electrical resistance without increasing the nanowire mass used. Several approaches such as laser sintering^27–29^, plasma processing^30^, and electron beam processing^31^ have been developed to perform nanowire fusion but are limited by their low throughput, high complexity, considerable environmental obstacles (e.g. vacuum conditions). Oven-based sintering of such nanowire-fabrics has been demonstrated, but requires the coated fabric to be heated to high temperatures for relatively long durations (e.g., 100 °C for 3 minutes^32^) that can damage the fabrics. We further note that the Ag nanowire-fabric patches also need to retain their performance under washing, under humid and high temperature environments, and under repeated mechanical bending^33,34^.

Scalable post-deposition processing methods that do not damage the fabric, while retaining the above patch performance characteristics, and reducing the mass of nanowires required, are needed to enable widespread deployment of these fabric-based personal heaters. Intense pulsed light (IPL) from a xenon lamp has been used to excite plasmon resonance induced heating and resulting sintering of metal nanowires on polymer substrates under ambient conditions, within seconds, and without damaging the substrate^15,35–39^. Further, the large area capability of such xenon lamps (e.g, optical footprint ≥ 12 inches × 1 inch in this work) lends itself to scalable manufacturing. However, these works have been limited to polymer substrates which have higher thermal damage thresholds and significantly different thermal properties than commonly worn woven fabrics. Printing and IPL sintering of metallic NPs on textiles to fabricate a solid-state fabric-based capacitor^40^ and RFID antennas^41^ has been demonstrated. However, the focus of these works was on characterizing device characteristics as a function of the printing parameters rather than on identifying the effect of IPL parameters and understanding how it affects durability of the fabric-based devices.

**Figure 1:**
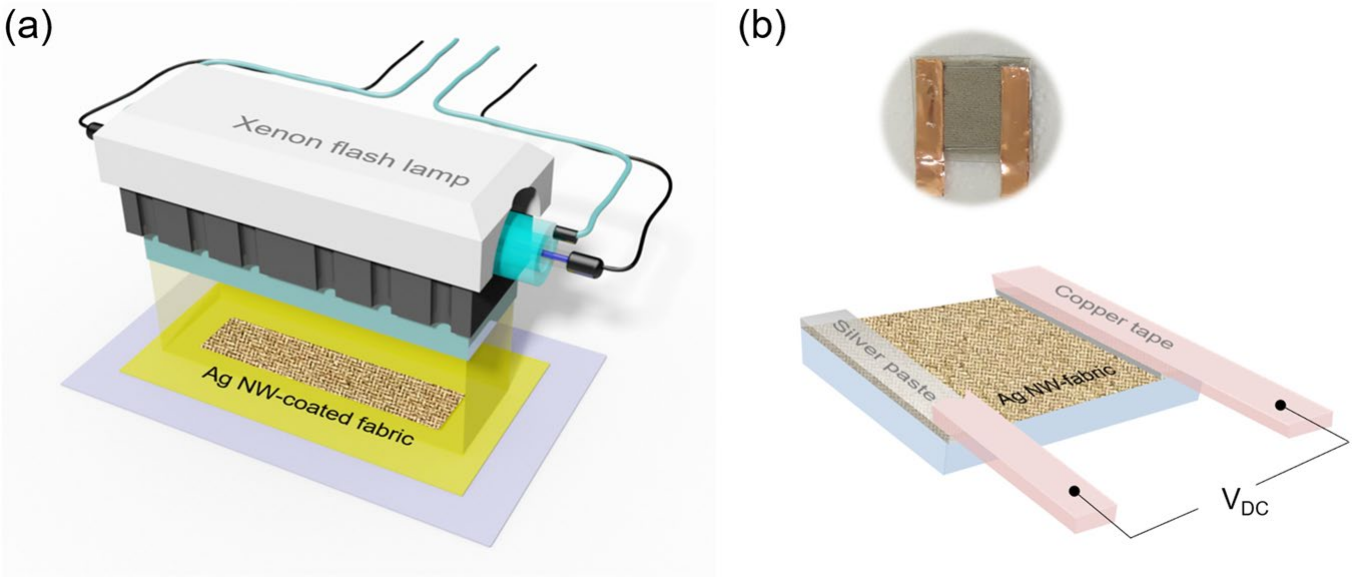
Schematic of (**a**) IPL sintering system for Ag nanowire-fabrics and (**b**) the Ag nanowire-fabric with silver paste copper tape adhered to the fabric using silver paste, for applying DC voltage and measuring resistance of the fabric patch.

This paper investigates the effect of IPL sintering (Fig. 1a) on the electrical, thermal, microstructural and durability characteristics of Ag nanowire on woven polyester heating patches (Fig. 1b). Ag nanowires are deposited on the fabrics using a simple dip-and-dry method. The number of dip-coating cycles is varied, to investigate the effect of added Ag nanowire mass on the performance of the patch. The IPL irradiance and pulse duration are varied to identify the optimum IPL parameters that minimize the electrical resistance of the patch. The effect of the IPL parameters and added mass of Ag nanowires on the micromorphology and crystallinity of the sintered nanowires is characterized via Scanning Electron Microscopy and X-Ray Diffraction. The stability of the IPL-sintered Ag nanowire-fabrics under mechanical, washing, and environmental tests is compared to that of conventionally fabricated nanowire-fabrics. The degree and repeatability of temperature rise of the patch under applied voltage is measured using an infrared camera. It is shown that IPL sintering results in concurrently greater thermal performance and durability of the patch, with lower mass of Ag nanowires than conventional dip-and-dry approaches. Multi-physical modeling is performed to estimate nanoscale optical absorption, temperature gradients on the polyester fibers, and fusion of the nanowires to understand why IPL sintering creates the above advantages.

## Results

### Electrical Conductivity and Microstructure

Dip-coating was employed as a nanowire deposition technique due to its simplicity and reproducibility (details in Methods section). This process resulted in the surface of the polyester fabric being coated with Ag nanowires, driven by capillary forces generated at the fabric-liquid interface during solvent evaporation^42^. The number of dip coating cycles was varied from one to five, effectively changing the added nanowire mass. Henceforth the number of dip coating cycles is referred to using “Dip-” followed by the number of coating cycles. For example, Dip-5 denotes five dip coating cycles and Dip-3 denotes 3 dip coating cycles. Optical images of the nanowire-fabric patches (Supplementary Fig. S1a) show that the fabric color turned from a splotchy and non-uniform light gray to a more uniform black-gray with greater dip-coating cycles. This is because the fabrics were only partially stained by the Ag nanowires ink when the number of dip-coating cycle was lesser than 3^42,43^, but were coated uniformly at and beyond 3 dip-coating cycles. As expected, the sheet resistance of the Ag nanowire-fabric decreased with increasing dip-coating cycles (Supplementary Fig. S1a) to 0.71 ohm·sq^−1^ for Dip-3 as compared to Dip-1. However, the resistance did not decrease as significantly from 3 to 5 cycles. This dependence of patch resistance on added nanowire mass has been observed in past work as well^44^. The lowest as-deposited sheet resistance of 0.66 ohm·sq^−1^ was achieved after 5 dip-coating cycles. This reduction in resistance is accompanied by an increase in the added mass of Ag nanowires (Supplementary Fig. S1a) from 0.06 mg·cm^−2^ for Dip-1 to 0.33 mg·cm^−2^ for Dip-5 samples. SEM images of the as-deposited patches show individual polyester fibers visible for low added nanowire mass (Dip-1, Supplementary Fig. S1b)) while greater added nanowire mass (Dip-3 and Dip-5, Supplementary Fig. S1c,d) results in a loss of visible distinction between individual fibers at the mesoscale due to being covered up with nanowires. At the same time, the mesoscale definition of the weave itself is preserved. Since a uniform coating is obtained after 3 dip coating cycles, IPL sintering and property characterization were performed only for Dip-3 and Dip-5 patches. During IPL sintering the optical irradiance was changed from 6 to 16 kW/cm^2^ by changing the voltage from 2 to 3 kV and changing the pulse duration from 100 µs to 500 µs (Table 1). Further details on the experimental aspects of IPL sintering are provided in the Methods section, along with details on the post-IPL characterization and testing.

**Table 1:** IPL parameters used in experiments.

Lamp voltage [kV]	Irradiance [kW/cm^2^]	Pulse duration [µsec]	Fluence [J/cm^2^]
2.0	6	100	0.59
		200	1.19
		300	1.78
		400	2.37
		500	2.97
2.5	10	100	1.01
		200	2.03
		300	3.04
		400	4.06
		500	5.07
3.0	16	100	1.57
		200	3.14
		300	4.71
		400	6.28
		500	7.85

**Figure 2:**
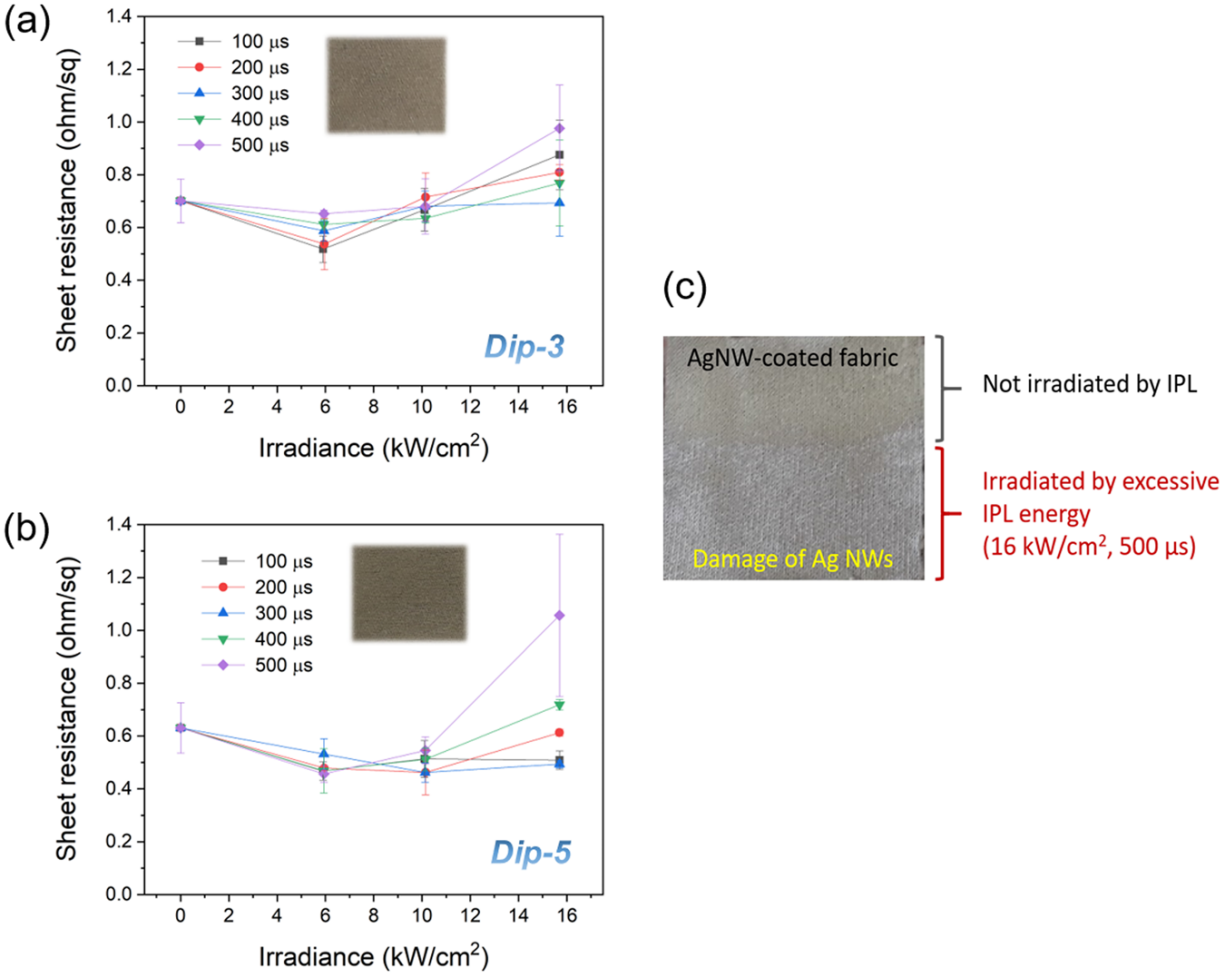
Electrical properties of IPL sintered Ag nanowire-fabrics. Sheet resistance of IPL sintered Ag nanowire-fabrics for different irradiance and pulse duration for (**a**) Dip-3 (**b**) Dip-5 cases. (**c**) Optical image of Ag nanowire-fabric half exposed to IPL irradiance 16 kW·cm^−2^ for 500 µs pulse duration.

**Figure 3:**
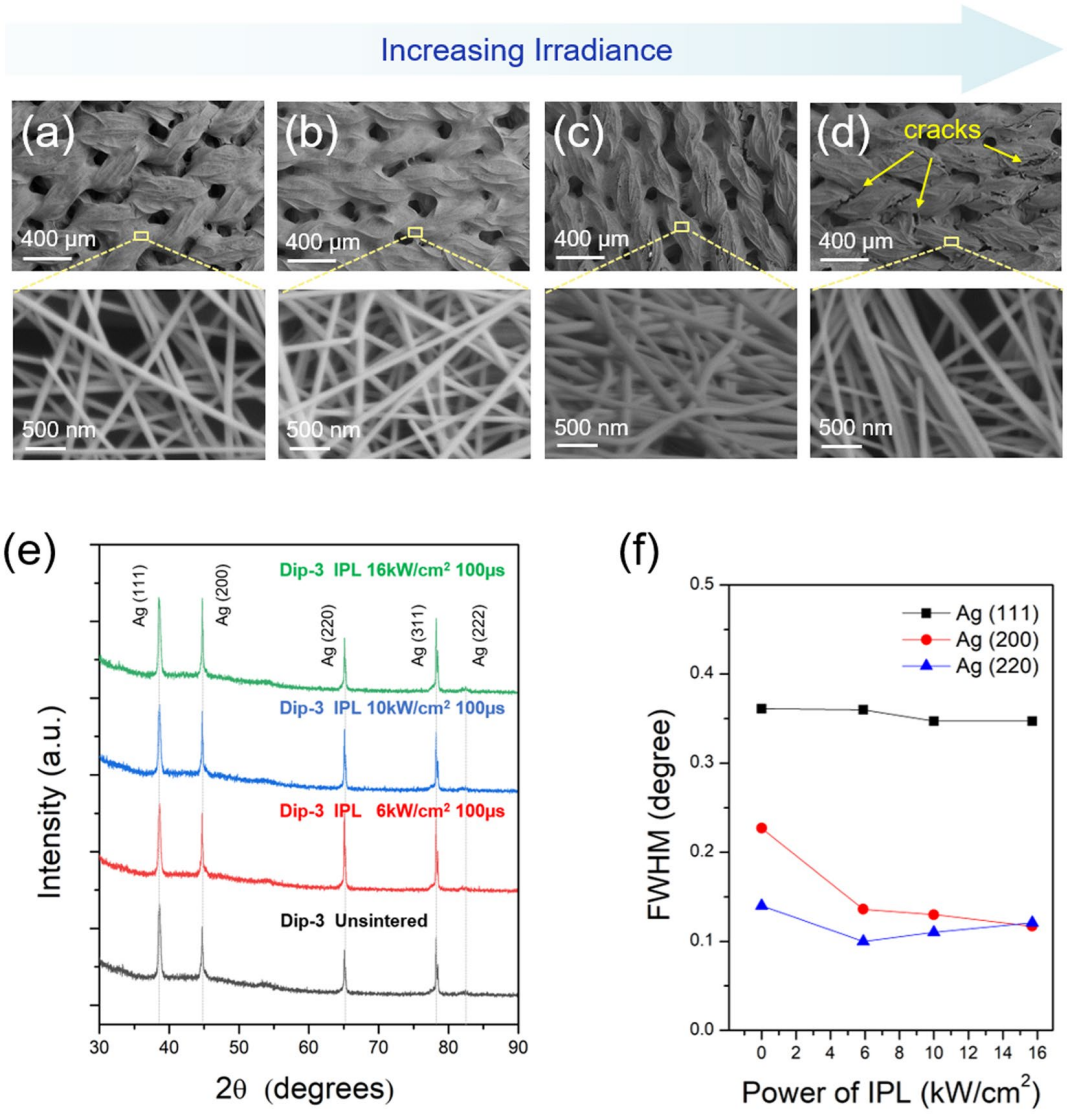
Characterization of Dip-3 Ag nanowire-fabrics. SEM images (**a**–**d**) and the XRD spectra (**e**) of the Ag nanowire-fabrics for pulse duration 100 µs and irradiance (**a**) 0 kW·cm^−2^ (unsintered), (**b**) 6 kW·cm^−2^, (**c**) 10 kW·cm^−2^, and (**d**) 16 kW·cm^−2^; (**f**) The full-width at half maximum (FWHM) for Ag (111), (200), and (220) XRD peaks with the same pulse duration as above.

**Figure 4:**
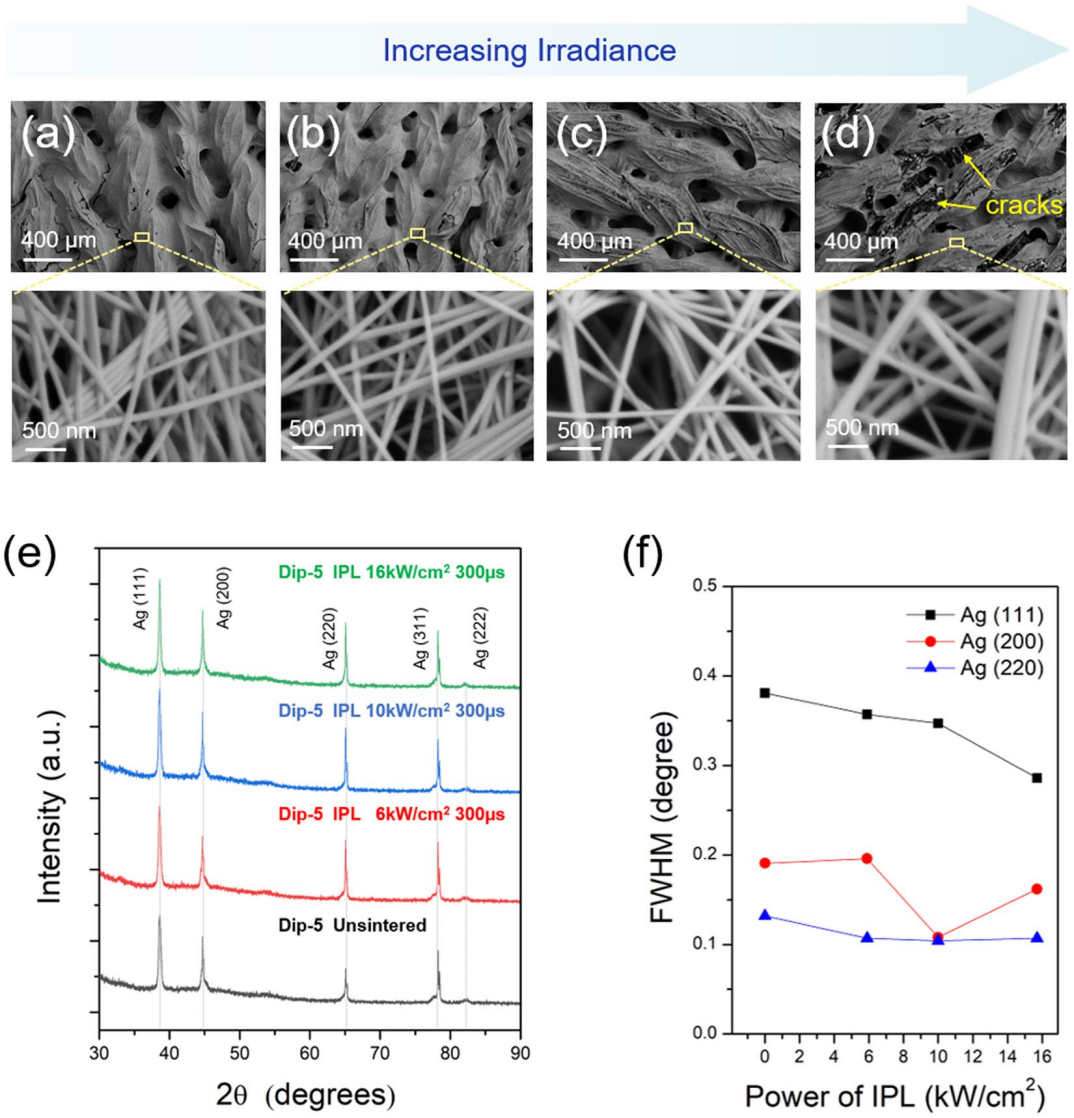
Characterization of Dip-5 Ag nanowire-fabrics. SEM images (**a**–**d**) and the XRD spectra (**e**) of the Ag nanowire-fabrics for pulse duration 300 µs and irradiance (**a**) 0 kW·cm^−2^ (unsintered), (**b**) 6 kW·cm^−2^, (**c**) 10 kW·cm^−2^, and (**d**) 16 kW·cm^−2^; (**f**) The full-width at half maximum (FWHM) for Ag (111), (200), and (220) XRD peaks with the same pulse duration as above.

Figure 2 compares the sheet resistance of the IPL sintered Ag nanowire-fabrics to that of the as-deposited fabric (shown as 0 irradiance). For Dip-3 samples, the post-IPL sintering sheet resistance is lowest at 6 kW·cm^−2^ irradiance (Fig. 2a). However, for irradiance 10 kW·cm^−2^ or higher the resistance increases again. An explanation for this phenomenon is obtained from SEM images and XRD spectra. When Ag nanowires are exposed to IPL light surface plasmon resonance results in absorbed photons being converted to heat, resulting in rise in nanowire temperature and fusion between the Ag nanowires at their contacts^35,45^. The XRD spectra showed that the full-width at half-maximum (FWHM) of Ag (200), and (220) peaks (JCPDS No. 04-0783) reduces after IPL at irradiance of 6 kW·cm^−2^ and higher (Fig. 3e,f), indicating that the Ag nanowires are being fused. This is why IPL at 6 kW·cm^−2^ irradiance reduces the resistance. SEM images (Fig. 3c,d) show that at irradiance higher than 6 kW·cm^−2^ leads to gaps in the mesoscopic Ag nanowire network which are likely responsible for the increase in electrical resistance. The corresponding XRD spectra still show a reducing FWHM for the Ag (111) and (200) peaks, indicating that nanowire fusion is still occurring at these higher irradiances. These mesoscale gaps in the nanowire network could be due to evaporation of the nanowires under excessive IPL irradiance. Figure 2c shows an optical image of an Ag nanowire-fabric exposed to IPL fluence of 16 kW·cm^−2^ with a pulse duration of 500 µs over approximately half of its area, while the remainder is left unexposed. The nanowire evaporation is clearly demonstrated by observing that the underlying white color of the fabric is partially exposed in the region exposed to IPL. For the Dip-5 case, the sheet resistance decreases with IPL irradiance up to 10 kW·cm^−2^ (Fig. 2b). Again, the reduction in the FWHM of the Ag (111), (200), and (220) peaks till 10 kW·cm^−2^ irradiance (Fig. 4e,f) supports nanowire-fusion enabled reduction in resistance. The occurrence of large mesoscale gaps in the nanowire network (Fig. 4d) at higher irradiance explains the increase in resistance due to nanowire evaporation beyond the above optimal irradiance. Note that the SEM images also show that the average distance between the nanowires is significantly lesser than the peak wavelength of radiative loss from the human body (≈9 µm), indicating that the coated fabrics will concurrently reduce heat loss from the body^7^.

The optimal IPL sintering parameters for Dip-3 samples were 100 µs pulse duration with 6 kW·cm^−2^ pulse irradiance (fluence 0.59 J·cm^−2^), resulting in a lowest achievable sheet resistance of 0.51 ohm·sq^−1^ and a resistance reduction of 28% after IPL (Fig. 2a). The optimal IPL sintering parameters for Dip-5 samples were 10 kW·cm^−2^ irradiance and 300 µs duration (fluence 3.04 J·cm^−2^), resulting in the lowest resistance of 0.46 ohm·sq^−1^ (Fig. 2b) and a resistance reduction of 30% after IPL. Since temperature rise and resultant nanowire fusion in IPL sintering is via optically-induced heating, greater amount of nanowire material requires greater heat generation for achieving the activation energy necessary for nanowire fusion. Since the mass of added nanowires is greater for Dip-5 samples than for the Dip-3 samples (Supplementary Fig. S1a) the optimal fluence for the Dip-5 samples is also greater than that for the Dip-3 samples.

### Mechanical, Environmental and Wash Testing

**Figure 5:**
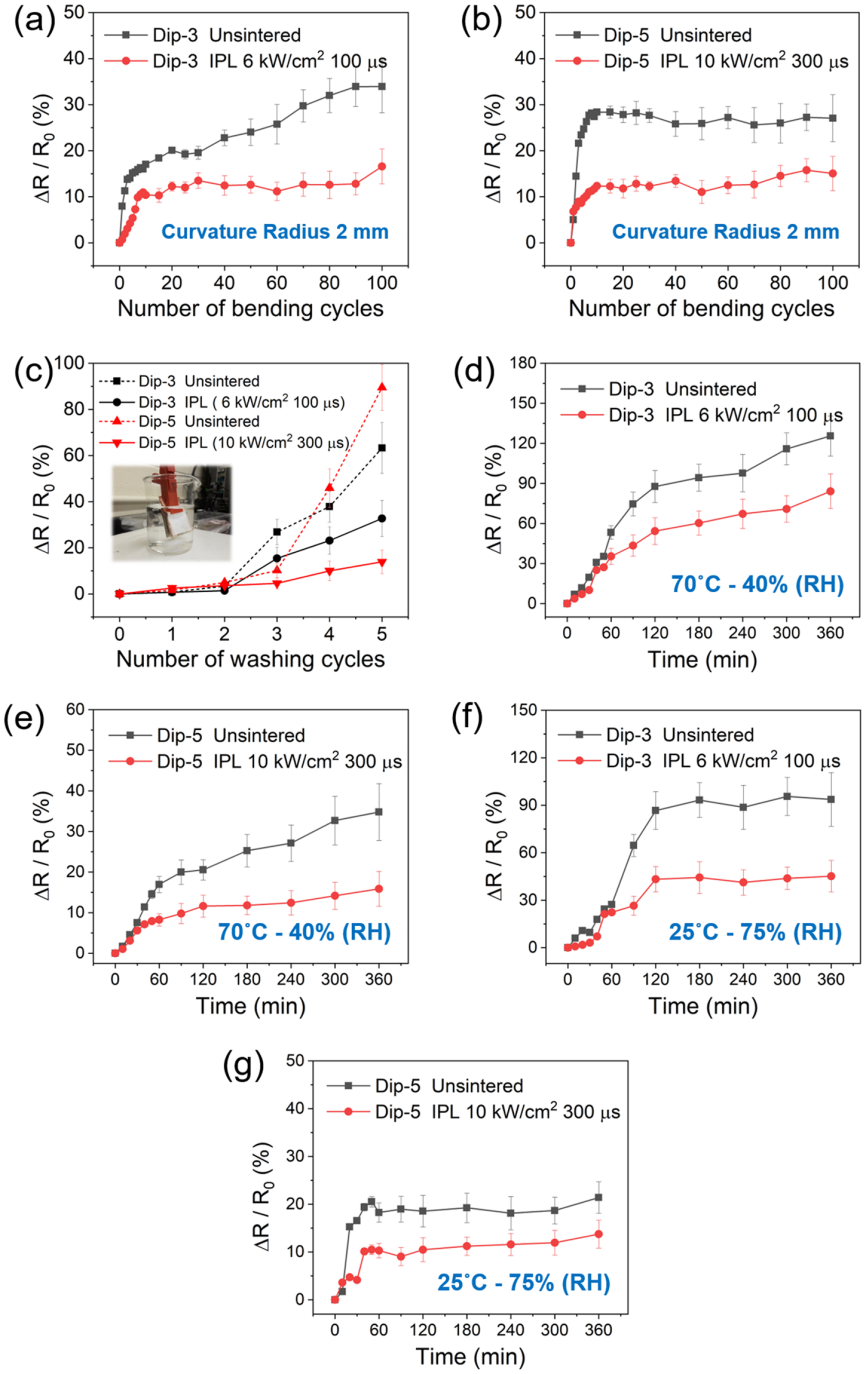
Stability test. The normalized change in sheet resistance during bending test of as-deposited and IPL sintered (**a**) Dip-3 samples and (**b**) Dip-5 samples. (**c**) The normalized change in sheet resistance after washing test for Dip-3 (black) and Dip-5 (red) Ag nanowire-fabrics before (dotted line) and after (solid line) IPL irradiation. Inset shows the washing test setup. The evolution of normalized change in sheet resistance for the Ag nanowire-fabrics before- (black) and after- (red) IPL irradiation under different temperature and humidity conditions; (**d**) Dip-3, 70 °C-40% RH, (**e**) Dip-5, 70 °C-40% RH, (**f**) Dip-3, 25 °C-75% RH, and (**g**) Dip-5, 25 °C-75% RH. (Dip-3 IPL parameters: 6 kW·cm^−2^, 100 µs; and Dip-5 IPL parameters: 10 kW·cm^−2^, 300 µs).

The mechanical bending tests, environmental reliability tests and wash tests were conducted on the optimally IPL sintered Ag nanowire-fabrics since they have the least electrical resistance. We use the normalized change in resistance *ΔR*/*R*_0_ as an estimate of performance stability of the patch. Here *R*_0_ is the as-deposited or IPL sintered sheet resistance and *ΔR* is the difference between the post-bending sheet/post-washing/post-environmental test sheet resistance and *R*_0_. Supplementary Fig. S2 shows a photograph of the mechanical testing setup and the displacement history that the nanowire-fabrics were subjected to. The *ΔR*/*R*_0_ of the as-deposited Dip-3 nanowire-fabrics increased by 13% after just 3 bending cycles, followed by an increase all the way up to 35% at 80 cycles, after which the *ΔR*/*R*_0_ reaches a steady state (Fig. 5a). This indicates a gradual weakening of Ag nanowire contacts causing an increase in the electrical resistance with bending, in the as-deposited Dip-3 sample. The *ΔR*/*R*_0_ of the IPL sintered nanowire-fabric stabilizes to a much lower value of around 12% after only the 20^th^ bending cycle. Again, for the Dip-5 Ag nanowire-fabric the *ΔR*/*R*_0_ of the as-deposited and IPL sintered patch stabilized to around 24–28% and 10–15% respectively after the 10^th^ bending cycle (Fig. 5b). Thus, IPL sintering significantly reduces the rise in resistance of the nanowire-fabrics under repeated mechanical loading.

In washing tests (Fig. 5c) the *ΔR*/*R*_0_ of the as-deposited and IPL sintered Ag nanowire-fabrics is stable until the second washing cycle. Starting from the third washing cycle, the *ΔR*/*R*_0_ for the as-deposited Dip-3 and Dip-5 samples increases with number of washing cycles to a maximum of 65% and 90% respectively. On the other hand, the increase in *ΔR*/*R*_0_ for the IPL sintering samples was remarkably lesser (30% and 12% for Dip-3 and Dip-5 samples respectively), clearly showing the higher durability of the IPL sintered nanowire-fabrics under washing. Further, the optimally sintered Dip-5 samples showed a significantly slower rate of increase in *ΔR/R*_0_ as compared to the optimally sintered Dip-3 samples. At an elevated temperature of 70 °C and 40% RH, the *ΔR/R*_0_ increased by 126% and 35% for as-deposited Dip-3 and Dip-5 samples respectively (Fig. 5d,e). The increase in *ΔR/R*_0_ was significantly lesser for the IPL sintered samples, i.e., 84% and 16% increase for Dip-3 and Dip-5 samples respectively. Again, the rate of increase in *ΔR/R*_0_ was much lesser for the Dip-5 samples than for the Dip-3 samples. At 70% RH and room temperature, the resistance of the as-deposited samples increased rapidly, likely due to oxidation^33^ (Fig. 5f,g). However, IPL sintering improved the stability under humid conditions by nearly twice as much as compared to the as-deposited samples. For this higher humidity case, the rate of increase in *ΔR/R*_0_ was quite similar for both Dip-3 and Dip-5 IPL sintered samples. These results show that IPL sintering results in greater stability to mechanical, environmental, and washing stresses. Further, based on the rate of change of *ΔR/R*_0_ in the above tests, that the optimally IPL sintered Dip-5 samples exhibit even better stability than the optimally IPL sintered Dip-3 samples. Greater insight into this increased durability is provided by the results from computational modeling.

### Joule Heating Test

**Figure 6:**
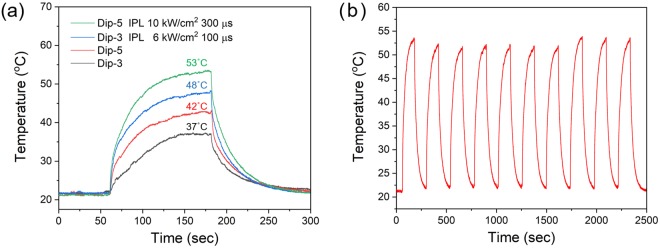
Joule heating test for the fabricated Ag nanowire-fabric patch. (**a**) Average temperature profile of the Ag nanowire-fabrics under 1 V applied voltage. (**b**) Average temperature response of optimally sintered Dip-5 patches over ten on/off cycles (2 min at on state and 2 min at off state) under 1 V applied voltage.

Figure 6a shows the evolution of average temperature of the as-deposited and IPL sintered nanowire-fabrics under 1 V applied voltage. The temperature increases rapidly upon applying voltage, until it reaches saturation at around 120 seconds due to balance of thermal losses with joule heating. A higher voltage of 1.2 V led to blackening of the fabric indicating fabric damage due to excessive heating. Since heat generation in the Ag nanowire-fabrics is due to Joule heating^9,10^, a lower sheet resistance leads to higher *Q*, which leads to higher steady-state temperature for the same applied voltage. IPL sintering enhances the Joule heating by enabling an increase in the *ΔT* of nearly 10 °C (26–30% increase) for both Dip-3 and Dip-5 samples (Fig. 6a). The maximum *ΔT* of 32 °C, reached for IPL-sintered Dip-5 Ag nanowire-fabric, is quite close to the required value of 35 °C for personal heating patches on garments^26^. Figure 6b shows the temperature response of the optimally IPL-sintered Dip-5 Ag nanowire-fabric, for ten repeated on/off voltage cycles with 2 minutes on time and 2 minutes off time. The heating and cooling response and the maximum saturation temperature are preserved with high repeatability, indicating high thermal stability of the IPL-sintered patches under cyclic operation.

We note that the reduction in sheet resistance from unsintered to optimally IPL sintered samples is around 30%, which results in the observed increase in steady-state-temperature during device operation. The durability tests show that for the optimally IPL sintered Dip-5 samples (highest conductivity achieved after IPL) the increase in sheet resistance after mechanical bending, high temperature test, high humidity test and washing tests is only around 10–15%, much lesser than the reduction as compared to the unsintered fabric enabled by IPL. Thus, the rise in temperature of the optimally IPL sintered Dip-5 patch after washing/heating/bending will still be higher than that of the unsintered patches which are not exposed to these conditions, and will definitely be higher than the unsintered patches that are exposed to these conditions.

### Nanoscale Optical and Thermal Modeling

**Figure 7:**
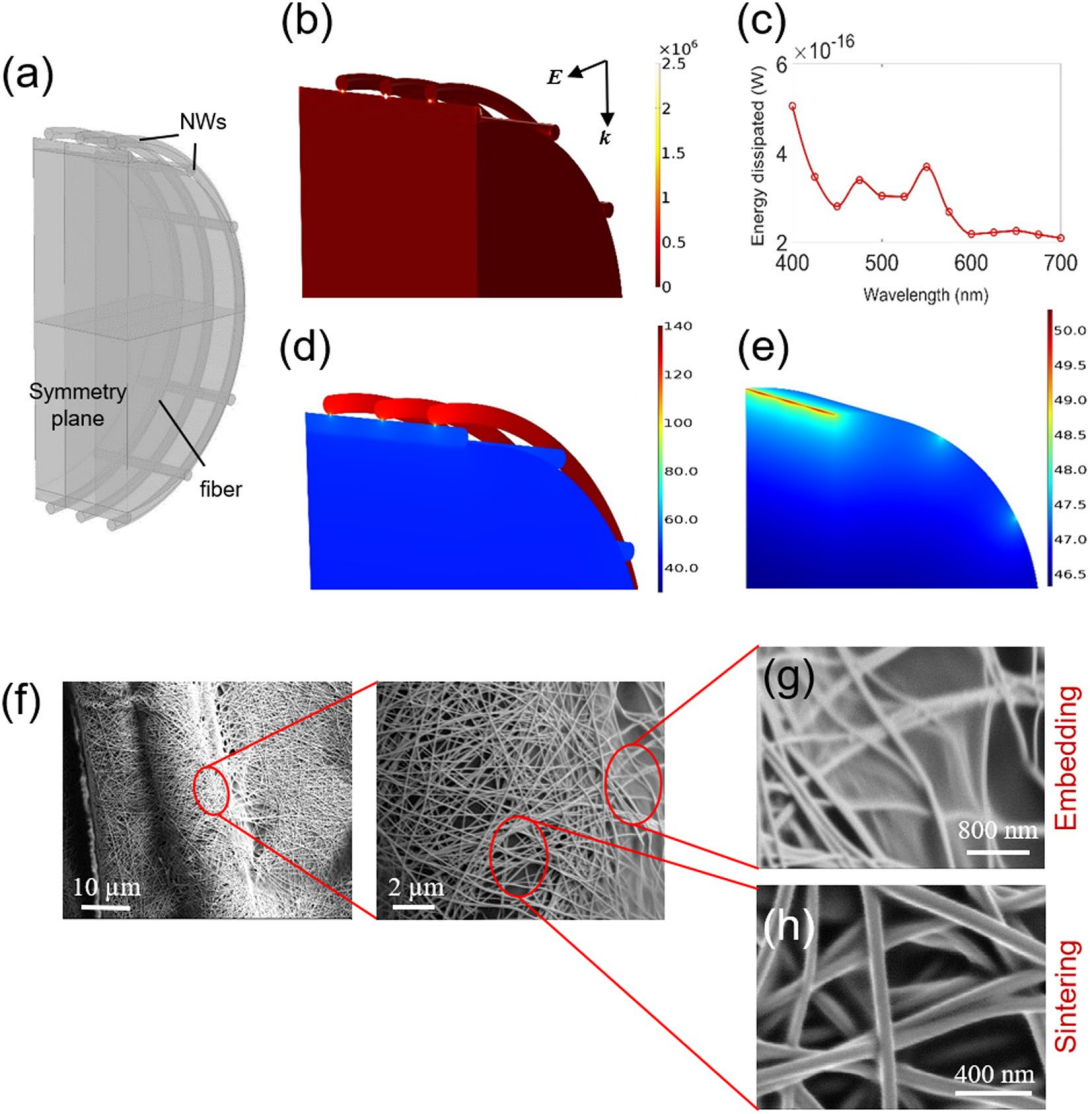
Optical and Thermal Modeling. (**a**) Schematic of modeled geometry. (**b**) Power dissipation density contours (in W/m^3^) for 450 nm wavelength and incident field intensity of 1 V/m. *E* shows direction of electric field polarization and *k* shows direction of wave propagation. (**c**) Calculated optical energy dissipated in the nanowires for a 1 V/m incident electric field. Calculated temperature contours (°C) at end of pulse duration for (**d**) nanowire-fiber ensemble and (**e**) for the fiber only. (**f**) SEM images of the Ag nanowire-fabrics for 10 kW/cm^2^ irradiance and 300 µs pulse duration. (**g**) Embedding of nanowires into the polyester fiber surface. (**h**) Sintering of nanowires at their contacts.

Finite Element Analysis (FEA) of optical absorption and heat transfer were combined to understand nanoscale temperature evolution during IPL sintering. A representative geometry consisting of Ag nanowires wrapped around a polyester fiber in two layers was considered (Fig. 7a) for optical and thermal simulations, to emulate nanowires coated on to the individual polyester fibers. The symmetry of the problem was used to model only half the geometry. Figure 7b shows the high energy dissipation density at the nanowire interfaces due to localized plasmon resonance. Hot spots were also observed at the nanowire-fiber interfaces but were significantly smaller than those at the nanowire-nanowire interfaces. The optical absorption spectrum (Fig. 7c) obtained by volume integration of the energy dissipation density shows two additional peaks besides the typical peak expected around 400 nm, likely due to the effect of the polyester fiber substrate.

This absorption spectrum was used, along with the manufacturer-specified energetic spectrum of the xenon lamp, to obtain total thermal dissipation for 10 kW/cm^2^ incident irradiance as described in previous work^46^. Since the nanowires have high aspect ratios and high thermal conductivities their Biot number is low and the effect of thermal conductivity within the nanowire assembly is negligible. Thus, the above obtained total thermal dissipation was assumed to be uniformly distributed for each nanowire and local temperature gradients in the nanowires were ignored. Conductive and convective losses were allowed from the nanowires and the fibers with the boundary conditions described in the Methods section. The thermal simulation was performed for pulse duration of 300 µs, corresponding to the optimal pulse irradiance and duration for the Dip-5 samples. Figure 7d show the temperature at the end of the pulse for the nanowire-fiber ensemble. The nanowires not in contact with the polyester fiber are at significantly higher temperature than the nanowires below it, with hot spots being created at the nanowire-nanowire interfaces. This is because the polyester fiber acts as a heat sink for the nanowires directly in contact with it, which is not the case for the other nanowires. Thus, a significant difference in temperature can be expected through the thickness of the nanowire coating, since the actual nanowire network does have much more than two layers of nanowires. The nanowires closer to the fabric will have lower temperatures than those above them due to the fiber acting as a heat sink. Figure 7e shows that the temperature at the fiber core only goes up to 46.5 °C and the maximum fiber temperature does not exceed 52 °C. A local radial temperature gradient is also created on the fiber surface around the nanowire-fiber contact points, with the peak temperature in this region matching that of the nanowires in contact with it. The maximum temperature gradient was at the nanowire-nanowire interface, of order of magnitude 10^10^ K/m, with a temperature gradient of 10^8^ K/m at the nanowire-fiber interface.

We also note that while a different direction of polarization will change the thermal absorption contours slightly (Supplementary Fig. S4), the low Biot number will result in insignificant temperature gradients in the nanowires themselves. Therefore, the location of the temperature gradients in the fabric will not change while the magnitude will. Further, since the nanowires are randomly oriented the polarization direction with respect to nanowire axes will change at different local spots on the fabric (likely including the configuration modeled here). The developed model does not capture all likely configurations of nanowires with respect to the incident field polarization but still does illustrate the phenomenon of local temperature gradients in the fabric.

This temperature profile also provides insight into the increase in durability of the nanowire-fabrics after IPL sintering. While the peak fiber temperature does not exceed its typical softening (glass transition) temperature the thermal expansion coefficient of polyester (≈123.5 K^−1^) is significantly greater than that of silver (≈19.5 K^−1^). Thus, during the IPL pulse the radial region of the fiber at the nanowire-fiber interface, that has the above mentioned radial temperature gradient, will expand by about 6 times more than the nanowire. The nanowires will locally sink, due to their weight, into the resulting trough temporarily created in the fiber surface. When the pulse is turned off the resulting thermal contraction will result in the nanowires being encapsulated partially by the surrounding contracting polyester material. Indications of this behavior can also be seen in SEM images in regions where the nanowire-fiber contact is exposed (Fig. 7g), which show that after IPL the nanowires seem to be partially embedded into the surrounding polyester while neck growth between nanowires happens simultaneously (Fig. 7h). Such localized embedding of nanowires into the underlying substrate is known to increase durability under mechanical loading^15,47,48^. The nanowire embedding will also reduce the active surface area of silver (which is more hydrophilic than polyester) available for degradation under humid, washing and high temperature conditions. This is why the durability of the IPL sintered fabrics is greater than that of the unsintered fabrics. Meanwhile, it was clearly observed that the nanowires not in contact with the fiber are fused at their contacts (Fig. 7h), corresponding to the result of the optical-thermal simulation which shows the hot spots are created at the nanowire-nanowire interfaces (Fig. 7b,d). Thus, the simulations are in good agreement with the experimental results.

## Discussion

This work demonstrates ultra-high speed intense pulsed light (IPL) sintering of Ag nanowires on woven polyester to fabricate highly electrically conductive Ag nanowire-fabrics usable as joule heated patches for the personal thermal management. We show that IPL sintering improves the electrical conductivity of the Ag nanowire-fabric from the dip-coated state, resulting in a lowest sheet resistance of 0.46 ohm·sq^−1^. IPL sintering also significantly increases the device durability under mechanical bending, washing, high temperature and high humidity conditions, despite the absence of any additional coating on the nanowire network. The IPL sintered samples undergo nearly 26–30% greater temperature rise as compared to the conventionally fabricated dip-and-dry nanowire-fabric during joule heating, at a low applied voltage of 1 Volt. This performance is met by the optimally sintered Dip-5 samples, as discussed in the experimental results section. Further, for these optimally sintered fabrics the added nanowire mass was only 0.33 mg·cm^−2^. The optimum IPL sintering was performed here at room temperature and under ambient conditions in just 300 microseconds (Dip-5 case).

We note that the thermal performance of the patch is not dependent just on the increase in temperature per unit voltage but also on the area over which this temperature rise occurs. A higher temperature rise (*∆T*) per unit voltage (*V*) is desirable over the largest patch area (*A*) possible is desirable. Thus, we use the term *A∆T/V* as an index of thermal performance of the patch to compare the optimally fabricated patch in this work to literature (Table 2). For our fabrics, a higher temperature rise can be achieved over a larger patch at a similar voltage resulting in a 70% higher index of performance than in the state-of-the-art. Further, within the scope of the data available in literature for Ag nanowire-fabrics, the added nanowire mass in this work is only half of that in previous work^7^. Thus, IPL sintering reduces the cost of the personal thermal management patch while increasing the device performance and increasing the durability under mechanical, washing, and environmental stresses without any additional coatings.

**Table 2:** Comparison of performance of heating fabrics. Dash indicates that data was not provided.

Nanomaterial & Fabric	Patch Size [cm^2^]	Resistance [ohm/sq]	Thermal Power [kW/cm^2^]	Nanowire Mass [mg/cm^2^]	*ΔT* [°C]	*A(ΔT/V)* [cm^2^°C/V]	Ref.
CNT, Cotton	4	5000	0.04	—	20	4	^13^
CNT, Cotton	6.5	—	—	2.9	34	14.3	^7^
Ag nanowire, Cotton	6.5	—	—	0.6	31	169	^7^
Ag nanowire, Nylon	6.3	1	0.36	—	35	145	^32^
Ag nanowire, Cotton	4	7.13	0.04	—	33	132	^44^
This Work	9	0.46	0.24	0.33	32	288	

The experimental observations in this work show that there is a fairly narrow window of pulse irradiance within which IPL sintering reduces the nanowire network resistance, and that the resistance also depends on the IPL pulse duration and the added nanowire mass. We also observe that excessive irradiance and pulse duration can reduce conductivity, due to evaporation of the Ag nanowires. The optical-thermal modeling provides greater insight into the process, indicating that there can be a significant difference in temperature through the thickness of the nanowire coating and radial temperature gradients at the nanowire-fiber interfaces. Based on the observed temperatures, a differential thermal expansion based nanowire embedding mechanism is proposed to explain the increased durability observed after IPL sintering. Since the IPL sintering process can be performed over an area of 12 inches by 1 inch in the same time, and without any additional changes to the setup, the throughput of IPL sintering in this work is limited only by the size of the sample used here and not by an inherent characteristic of the process. In fact, the compatibility of IPL sintering with large-area roll-to-roll deposition has been previously demonstrated^49^. Thus, the IPL sintering technique has the potential to be a key technology for scalable and cost-effective mass manufacturing of Ag nanowire-fabric heating patches that can be easily integrated with readymade garments.

## Methods

### Material preparation and fabrication of Ag nanowire-fabrics

Woven polyester fabric patches measuring 30 mm × 30 mm (500 μm thickness) were cleaned using ethanol (99.5%). Dip-coating was employed as a nanowire deposition technique, due to its simplicity and reproducibility. Ag nanowires (100 nm in diameter, 100–200 μm in length; ACS materials) were dispersed in ethanol to form a 2 mg·ml^−1^ suspension. The cleaned polyester fabric patches were vertically immersed into the Ag nanowire solution for 2 minutes and then dried at room temperature for 30 minutes. This process resulted in the surface of the polyester fabric being coated with Ag nanowires, driven by capillary forces generated at the fabric-liquid interface during solvent evaporation^42^. The number of dip coating cycles was varied from one to five, effectively changing the added nanowire mass. The quantity of added nanowire mass was obtained by measuring the change in mass of the nanowire-fabric after every coating cycle.

### Intense pulsed light sintering of Ag nanowire-fabrics

The above prepared Ag nanowire-fabric patches were exposed to IPL at room temperature and under ambient environmental conditions outside a chamber (Fig. 1a). The IPL system used here (Sinteron 3000, Xenon Corp., USA) consists of a xenon flash lamp with a reflector, a power supply and a pulse controller. The optical power from the lamp is distributed over a broad spectrum from 350 nm to 800 nm. The dip-coated samples were placed on a sample stage at 1 inch distance from the lamp, such that the optical footprint of the pulsed light at the substrate was approximately 12 inches × 1.2 inches. The optical energy output per pulse from the lamp (*E*_*p*_) is controlled by changing the pulse duration *t*_*on*_ (microseconds) and the lamp voltage *V*_*L*_ (volts), as per the relationship supplied by the lamp manufacturer *E*_*p*_ = (*V*_*L*_/3120)^2.4^ × *t*_*on*_. In this work, the number of IPL pulses was fixed at one. The IPL irradiance was changed from 6 to 16 kW/cm^2^ by changing the voltage from 2 to 3 kV and changing the pulse duration from 100 µs to 500 µs (Table 1).

### Characterization

The surface microstructure of the Ag-fabrics was examined via scanning electron microscopy (SEM) and the changes in crystal structure identified using X-ray diffraction (XRD, CuK radiation). To evaluate electrical conductivity copper tape electrodes were adhered to the Ag nanowire-fabric, using silver paste to minimize the contact resistance between the electrodes and the nanowires (Fig. 1b). The resistance across the electrodes was then measured using a Keithley 2400 C source meter. The mechanical stability of the nanowire-fabrics was investigated via cyclic bending to a radius of 2 mm, with the resistance change measured every cycle in the first 10 cycles and then every 10 cycles till a total of 100 cycles. The mechanical tester consisted of two aluminum clamps with insulating glass and a motorized stage (Thorlabs; LTS150). One clamp was fixed while the other was attached to the motorized stage. The fabric was initially kept straight. During the test, the stage moved cyclically by a displacement of 5 mm, causing the fabric to bend repeatedly. To simulate washing, the Ag nanowire-fabrics were stirred at 500 rpm in distilled water at 30 °C for 20 min and the change in resistance after multiple washing cycles was measured after the samples were dried. Environmental testing of the nanowire-fabrics was performed in an environmental chamber (Folyon H-300) at temperature and relative humidity (RH) of 25 °C-75% RH and 70 °C-40% RH, to capture changes in patch resistance induced by both ambient temperature-high humidity and high temperature-low humidity conditions. To evaluate the heat generation capacity of the patches, a constant DC voltage of 1 V was applied (using a power supply Protek; 18020 M) across the copper electrodes of each nanowire-fabric for 120 seconds, while using an infrared camera (Micro-Epsilon; TIM400) to measure temperature evolution.

### Modeling

The wave optics module in COMSOL was used to perform optical FEA simulations under the harmonic assumption^50,51^ to predict optical energy dissipation in the Ag nanowire and polyester fiber ensembles (Supplementary Fig. S3-a). The diameters for Ag nanowires and polyester fiber were 100 nm and 15 µm, respectively. The fiber diameter was obtained from SEM images. The ensemble was embedded in a layer of air, surrounded by a perfectly matched layer (PML) with a scattering boundary condition on the outer surfaces. The incident electric field had a magnitude 1 V/m. The electric field polarization was along to the axis of the top layer nanowire to minimize the coupling of the optical field into the nanowire ensemble, as a conservative condition^52^. The wavelength dependent real and imaginary refractive indexes of Ag were obtained from Johnson and Christy’s work^53^. The refractive index of the polyester was fixed at 1.5. The mesh sizes, size of air shell and size of the perfectly matched layer were refined till their effect on the predicted absorption and scattering curves was negligible. Perfect electric conductor boundary conditions were used at the air-PML interfaces that were normal to the electric field polarization direction. The thermal model consisted of the geometry shown in Supplementary Fig. S3-b with symmetric boundary conditions as shown. Convective losses were specified from all surfaces, except the symmetric boundaries, with convective loss coefficient of 10 W/m^2^-K.

## Electronic supplementary material

Supplementary Information

## Data Availability

The datasets generated during and/or analyzed during the current study are available from the corresponding author on reasonable request.

## References

[1] Beerepoot, M. & Marmion, A. Policies for Renewable Heat: An integrated approach *OECD Publishing: Paris*, **9** (2012).

[2] Zheng Y (2010). Biomass energy utilization in rural areas may contribute to alleviating energy crisis and global warming: A case study in a typical agro-village of Shandong, China. Renewable and Sustainable Energy Reviews.

[3] Foster G, Rahmstorf S (2011). Global temperature evolution 1979–2010. Environmental Research Letters.

[4] Bhat NV, Seshadri DT, Nate MM, Gore AV (2006). Development of conductive cotton fabrics for heating devices. Journal of Applied Polymer Science.

[5] Hao L (2012). Development and characterization of flexible heating fabric based on conductive filaments. Measurement.

[6] Wang S, Li Y, Hu J, Tokura H, Song Q (2006). Effect of phase-change material on energy consumption of intelligent thermal-protective clothing. Polymer Testing.

[7] Hsu P-C (2014). Personal thermal management by metallic nanowire-coated textile. Nano letters.

[8] Doganay D, Coskun S, Genlik SP, Unalan HE (2016). Silver nanowire decorated heatable textiles. Nanotechnology.

[9] Song T-B (2014). Nanoscale joule heating and electromigration enhanced ripening of silver nanowire contacts. ACS nano.

[10] Maize K (2015). Super-Joule heating in graphene and silver nanowire network. Applied Physics Letters.

[11] Samad YA, Li Y, Alhassan SM, Liao K (2014). Non-destroyable graphene cladding on a range of textile and other fibers and fiber mats. Rsc Advances.

[12] Kongahge D, Foroughi J, Gambhir S, Spinks GM, Wallace GG (2016). Fabrication of a graphene coated nonwoven textile for industrial applications. RSC Advances.

[13] Ilanchezhiyan P (2015). Highly efficient CNT functionalized cotton fabrics for flexible/wearable heating applications. RSC Advances.

[14] Rahman MJ, Mieno T (2015). Conductive cotton textile from safely functionalized carbon nanotubes. Journal of Nanomaterials.

[15] Song C-H, Han CJ, Ju B-K, Kim J-W (2016). Photoenhanced Patterning of Metal Nanowire Networks for Fabrication of Ultraflexible Transparent Devices. ACS Applied Materials & Interfaces.

[16] Li Xiaoting, Hu Haibo, Hua Tao, Xu Bingang, Jiang Shouxiang (2018). Wearable strain sensing textile based on one-dimensional stretchable and weavable yarn sensors. Nano Research.

[17] Cui H-W, Suganuma K, Uchida H (2015). Highly stretchable, electrically conductive textiles fabricated from silver nanowires and cupro fabrics using a simple dipping-drying method. Nano Research.

[18] Zhou Changjie, Yang Yanqin, Sun Na, Wen Zhen, Cheng Ping, Xie Xinkai, Shao Huiyun, Shen Qingqing, Chen Xiaoping, Liu Yina, Wang Zhong Lin, Sun Xuhui (2018). Flexible self-charging power units for portable electronics based on folded carbon paper. Nano Research.

[19] Buldum A, Lu JP (2001). Contact resistance between carbon nanotubes. Physical Review B.

[20] Jiu J, Suganuma K (2016). Metallic Nanowires and Their Application. IEEE Transactions on Components, Packaging and Manufacturing Technology.

[21] Langley D (2013). Flexible transparent conductive materials based on silver nanowire networks: a review. Nanotechnology.

[22] Song J, Li J, Xu J, Zeng H (2014). Superstable transparent conductive Cu@ Cu4Ni nanowire elastomer composites against oxidation, bending, stretching, and twisting for flexible and stretchable optoelectronics. Nano letters.

[23] Kim C-L, Penkov OV, Shin D-G, Kim D-E (2016). Investigation of micro-abrasion characteristics of thin metallic coatings by *in-situ* SEM scratch test. International Journal of Precision Engineering and Manufacturing.

[24] Thanh, N. V. K. & Phong, N. T. P. In *Journal of Physics: Conference Series*. 012072 (IOP Publishing).

[25] Nateghi MR, Shateri-Khalilabad M (2015). Silver nanowire-functionalized cotton fabric. Carbohydrate polymers.

[26] Sorel S, Bellet D, Coleman JN (2014). Relationship between material properties and transparent heater performance for both bulk-like and percolative nanostructured networks. ACS nano.

[27] Han S (2014). Fast plasmonic laser nanowelding for a cu‐nanowire percolation network for flexible transparent conductors and stretchable electronics. Advanced materials.

[28] Spechler JA, Arnold CB (2012). Direct-write pulsed laser processed silver nanowire networks for transparent conducting electrodes. Applied Physics A.

[29] Hong S (2015). Selective laser direct patterning of silver nanowire percolation network transparent conductor for capacitive touch panel. Journal of nanoscience and nanotechnology.

[30] Wang R (2016). Plasma-induced nanowelding of a copper nanowire network and its application in transparent electrodes and stretchable conductors. Nano Research.

[31] Xu S (2005). Nanometer‐Scale Modification and Welding of Silicon and Metallic Nanowires with a High‐Intensity Electron Beam. Small.

[32] Kim C-L, Lee J-J, Oh Y-J, Kim D-E (2017). Smart wearable heaters with high durability, flexibility, water-repellent and shape memory characteristics. Composites Science and Technology.

[33] Wang X, Wang R, Shi L, Sun J (2015). Synthesis of metal/bimetal nanowires and their applications as flexible transparent electrodes. Small.

[34] Shi L (2015). A long-term oxidation barrier for copper nanowires: graphene says yes. Physical Chemistry Chemical Physics.

[35] Chung W-H, Kim S-H, Kim H-S (2016). Welding of silver nanowire networks via flash white light and UV-C irradiation for highly conductive and reliable transparent electrodes. Scientific reports.

[36] Chung Wan-Ho, Park Sung-Hyeon, Joo Sung-Jun, Kim Hak-Sung (2018). UV-assisted flash light welding process to fabricate silver nanowire/graphene on a PET substrate for transparent electrodes. Nano Research.

[37] Mallikarjuna K, Hwang H-J, Chung W-H, Kim H-S (2016). Photonic welding of ultra-long copper nanowire network for flexible transparent electrodes using white flash light sintering. RSC Advances.

[38] Song C-H (2015). Intense-pulsed-light irradiation of Ag nanowire-based transparent electrodes for use in flexible organic light emitting diodes. Organic Electronics.

[39] Li R-Z, Hu A, Zhang T, Oakes KD (2014). Direct writing on paper of foldable capacitive touch pads with silver nanowire inks. ACS applied materials & interfaces.

[40] Jang Y (2017). 2D all-solid state fabric supercapacitor fabricated via an all solution process for use in smart textiles. Applied Physics Letters.

[41] Björninen, T., Virkki, J., Sydänheimo, L. & Ukkonen, L. In *2015 Interna*tional *Conference on El*ectrom*agnetics in Advanced Applications (ICEAA)*. 589–592.

[42] Deegan RD (1997). Capillary flow as the cause of ring stains from dried liquid drops. Nature.

[43] Routh AF (2013). Drying of thin colloidal films. Reports on Progress in Physics.

[44] Yu Z, Gao Y, Di X, Luo H (2016). Cotton modified with silver-nanowires/polydopamine for a wearable thermal management device. RSC Advances.

[45] Staleva H (2009). Coupling to light, and transport and dissipation of energy in silver nanowires. Physical Chemistry Chemical Physics.

[46] Dexter M, Gao Z, Bansal S, Chang C-H, Malhotra R (2018). Temperature, Crystalline Phase and Influence of Substrate Properties in Intense Pulsed Light Sintering of Copper Sulfide NanoparticleThin Films. Scientific Reports.

[47] Jiu J (2012). Strongly adhesive and flexible transparent silver nanowire conductive films fabricated with a high-intensity pulsed light technique. Journal of Materials Chemistry.

[48] Zhong Z (2016). Roll-to-roll-compatible, flexible, transparent electrodes based on self-nanoembedded Cu nanowires using intense pulsed light irradiation. Nanoscale.

[49] Perelaer J (2012). Roll‐to‐Roll Compatible Sintering of Inkjet Printed Features by Photonic and Microwave Exposure: From Non‐Conductive Ink to 40% Bulk Silver Conductivity in Less Than 15 Seconds. Advanced Materials.

[50] Bansal S, Malhotra R (2016). Nanoscale-shape-mediated coupling between temperature and densification in intense pulsed light sintering. Nanotechnology.

[51] Dexter M, Bhandari R, Chang CH, Malhotra R (2017). Controlling processing temperatures and self-limiting behaviour in intense pulsed sintering by tailoring nanomaterial shape distribution. RSC Advances.

[52] Garnett Erik C., Cai Wenshan, Cha Judy J., Mahmood Fakhruddin, Connor Stephen T., Greyson Christoforo M., Cui Yi, McGehee Michael D., Brongersma Mark L. (2012). Self-limited plasmonic welding of silver nanowire junctions. Nature Materials.

[53] Johnson PB, Christy RW (1972). Optical Constants of the Noble Metals. Physical Review B.

