# Supplementary material for: Rapid Pulsed Light Sintering of Silver Nanowires on Woven Polyester for personal thermal management with enhanced performance, durability and cost-effectiveness
Source: Sci Rep. 2018 Nov 21;8:17159. doi: 10.1038/s41598-018-35650-7 (PMC6249281; doi:10.1038/s41598-018-35650-7)
Supplement: Supplementary file 1 — Supplementary Information [file 41598_2018_35650_MOESM1_ESM.pdf]

Supplementary Information

Rapid Pulsed Light Sintering of Silver Nanowires on Woven Polyester for personal thermal management with enhanced performance, durability and cost-effectiveness

Hyun-Jun Hwang, Harish Devaraj, Chen Yang, Zhongwei Gao, Chih-hung Chang, Howon Lee, and R. Malhotra*

1. Effect of the number of dip-coating cycles.

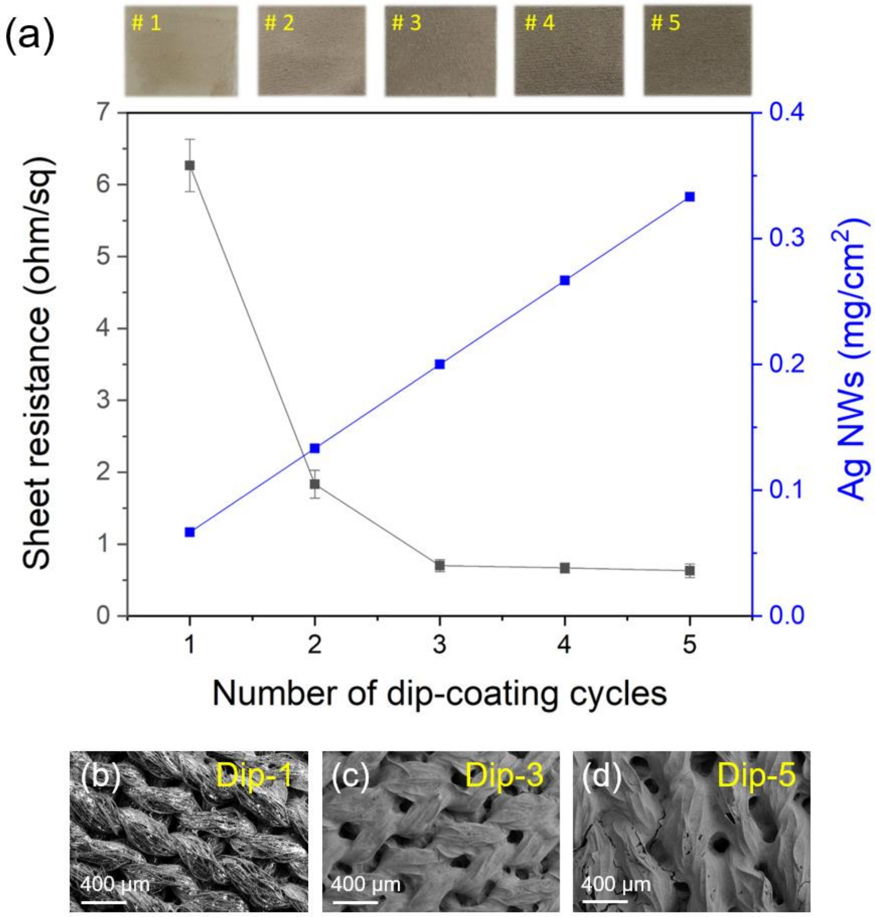

Figure S1. (a) The sheet resistance of the as-deposited Ag nanowire-fabrics as a function of the number of dip-coating cycles. SEM images of (b) Dip-1, (c) Dip-3, and (d) Dip-5 Ag nanowire-fabrics.

2. Mechanical bending stability.

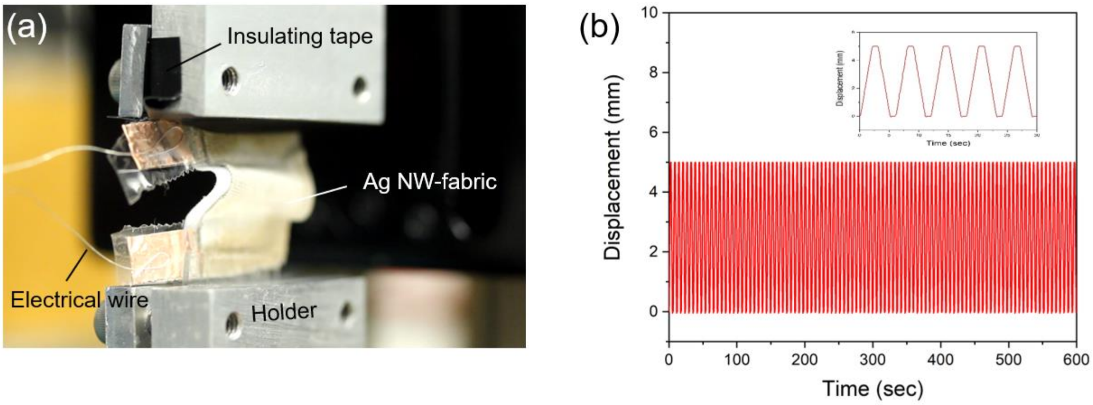

Figure S2. (a) Custom-made mechanical bending test setup and (b) displacement history during the bending test, inset shows displacement for 5 cycles.

3. Joule heating test for the fabricated Ag nanowire-fabric patch

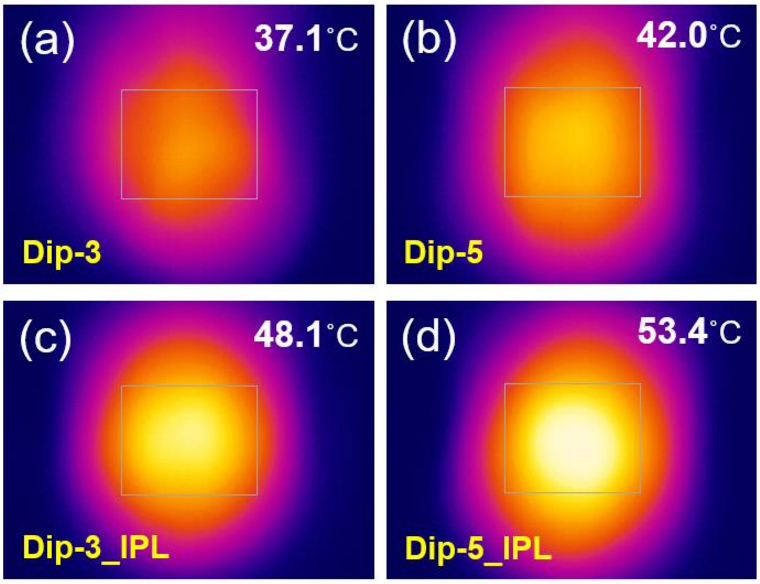

Figure S3. Infrared camera images of the Ag nanowire-fabrics at the saturation temperature for (a) as-deposited Dip-3 Ag nanowire-fabric, (b) as-deposited Dip-5 Ag nanowire-fabric, (c) IPL sintered Dip-3 Ag nanowire-fabric (IPL irradiance 6 kW·cm^{-2} and pulse duration of 100 μs), and (d) IPL sintered Dip-5 Ag nanowire-fabric (IPL irradiance 10 kW·cm^{-2} and pulse duration of 300 μs).

4. Optical and Thermal Modeling

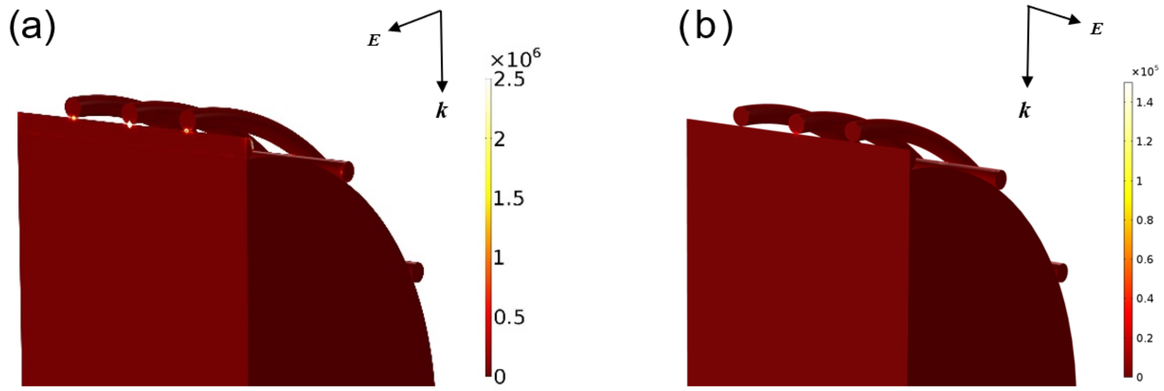

Figure S4. Effect of the polarization direction. Power dissipation density contours (in W/m^3) for 450 nm wavelength and incident field intensity of 1 V/m. *E* shows direction of electric field polarization and *k* shows direction of wave propagation. The electric field polarization (*E*) is perpendicular (a) and longitudinal (b) to the axis of the NW contacting with the fiber.
